# Epitope Dampening Monotypic Measles Virus Hemagglutinin Glycoprotein Results in Resistance to Cocktail of Monoclonal Antibodies

**DOI:** 10.1371/journal.pone.0052306

**Published:** 2013-01-03

**Authors:** Patrycja J. Lech, Gregory J. Tobin, Ruth Bushnell, Emily Gutschenritter, Linh D. Pham, Rebecca Nace, Els Verhoeyen, François-Loïc Cosset, Claude P. Muller, Stephen J. Russell, Peter L. Nara

**Affiliations:** 1 Department of Molecular Medicine, Mayo Clinic, Rochester, Minnesota, United States of America; 2 Biological Mimetics, Inc., Frederick, Maryland, United States of America; 3 INSERM, U758, Human Virology Laboratory, EVIR Team, Lyon, France; 4 Ecole Normale Supérieure de Lyon, Lyon, France; 5 Université de Lyon, UCB-Lyon1, Lyon, France; 6 Department of Immunology and WHO Collaborating Center for Reference and Research on Measles Infections, Centre de Recherche Public de la Santé/Laboratoire National de Santé, Luxembourg, Luxembourg; 7 College of Veterianty Medicine, Iowa State University, Ames, Iowa, United States of America; Wadsworth Center, New York State Dept. Health, United States of America

## Abstract

The measles virus (MV) is serologically monotypic. Life-long immunity is conferred by a single attack of measles or following vaccination with the MV vaccine. This is contrary to viruses such as influenza, which readily develop resistance to the immune system and recur. A better understanding of factors that restrain MV to one serotype may allow us to predict if MV will remain monotypic in the future and influence the design of novel MV vaccines and therapeutics. MV hemagglutinin (H) glycoprotein, binds to cellular receptors and subsequently triggers the fusion (F) glycoprotein to fuse the virus into the cell. H is also the major target for neutralizing antibodies. To explore if MV remains monotypic due to a lack of plasticity of the H glycoprotein, we used the technology of Immune Dampening to generate viruses with rationally designed N-linked glycosylation sites and mutations in different epitopes and screened for viruses that escaped monoclonal antibodies (mAbs). We then combined rationally designed mutations with naturally selected mutations to generate a virus resistant to a cocktail of neutralizing mAbs targeting four different epitopes simultaneously. Two epitopes were protected by engineered N-linked glycosylations and two epitopes acquired escape mutations via two consecutive rounds of artificial selection in the presence of mAbs. Three of these epitopes were targeted by mAbs known to interfere with receptor binding. Results demonstrate that, within the epitopes analyzed, H can tolerate mutations in different residues and additional N-linked glycosylations to escape mAbs. Understanding the degree of change that H can tolerate is important as we follow its evolution in a host whose immunity is vaccine induced by genotype A strains instead of multiple genetically distinct wild-type MVs.

## Introduction

Measles is the most contagious viral disease and remains one of the leading causes of death among young children globally. Although unvaccinated young children are at highest risk of measles and its complications, the disease can affect susceptible people of all ages. There is no specific treatment for measles and healthy people recover within 2–3 weeks, acquiring life-long immunity to the virus [80]. Despite a large number of clades and genotypes, current live-attenuated measles virus vaccines provide protection against all wild-type viruses because they alone belong to the single serotype.

The Measles virus spreads by aerosol droplets from coughing and sneezing and remains active and contagious in the air or on infected surfaces for up to two hours [80]. With a basic reproduction number of 12–15 the MV is the most contagious virus known, taking advantage of any lapse in vaccination effort in case of primary or secondary vaccine failure. Therefore outbreaks continue to occur, even in regions where the disease has previously been eliminated [1], [2], [3]. A number of factors have raised concerns that the virus may be subjected to considerable immune pressure [4], [5], [6]. It is therefore important to monitor MV strain evolution and understand the mechanisms that restrain the virus from escaping an individuals' immune response.

The MV is a negative strand, enveloped RNA virus of the genus morbillivirus within the family paramyxoviridae [7]. The viral hemagglutinin (H) glycoprotein is the major target for neutralizing antibodies [8]. Together with the fusion (F) glycoprotein it forms the hetero-oligomeric fusion complex to facilitate viral entry into the host cell [9], [10]. H protein attaches the virus to the target cell by binding to a cellular receptor and triggers the F protein to fuse viral and host cell membranes for entry [11]. The receptor-binding interface is located on the side of the H cuboidal head and makes contact with all known MV receptors [10], [12], [13], namely CD46 [14], [15], [16], [17], Signaling lymphocyte activation molecule (SLAM) [18], [19] and Nectin-4 (Polio virus receptor like protein, PVRL4) [20], [21]. It is also within an area targeted by neutralizing monoclonal antibodies (mAb) [12], [13]. It has thus been suggested that MV exists as a single serotype because the receptor-binding interface is highly unfavorable to mutation due to its functional importance [10], [12], [13].

The mutation rate of the measles virus in culture is similar to that of other RNA viruses and is estimated at 10^−5^ per base per replication [22]. This corresponds to about one mutation per RNA genome per replication. The combination of rapid replication kinetics and an elevated mutation rate is advantages to RNA viruses, as it allows them to quickly adapt to changes in their host environment (natural selection) [23]. For example, a single mutation resulting in an amino acid substitution can preclude a specific viral protein-antibody interaction or generate a potential N-linked glycosylation site (PNGS) available for post-translational oligosaccharide (glycan) attachment. Glycans are hydrophilic, branched chains of eight or more monosaccharides, which have the capacity to cover a substantial portion of the underlying protein surface and protect it from recognition by more than one antibody [24]. HIV-1 is a well studied example of a RNA virus that very successfully uses a dynamic glycan shield to protect the gp120 protein from immune recognition, with nearly 50% of the gp120 mass being due to carbohydrate residues attached to 20–25 identified PNGS [25], [26], [27], [28], [29]. In comparison, the MV H glycoprotein only has four conserved PNGS (N168, N187, N200 and N215), which are glycan modified [30]. Some of the MV clade D genotypes have acquired a fifth glycosylated PNGS following a D416N mutation [31]. It has not been explored why MV does not select for a more dynamic and extensive glycan shield. It is possible that extensive glycosylation at critical antigenic sites may be deleterious to viral fitness, such that it impairs protein folding or trafficking [30], hetero-oligomeric fusion complex formation and dynamics [9] or receptor binding [12], [13], [32], [33].

Our aim was to investigate if MV hemagglutinin glycoprotein can tolerate multiple escape mutations (including PNGS) in different epitopes and evade a polyclonal attack by multiple mAbs. We show that different mutations can be used to protect an epitope against a mAb. We ultimately generated a mutant MV with two rationally designed N-linked glycosylations and artificially selected point mutations that resisted neutralization by mAbs targeting four different epitopes simultaneously. Three of these epitopes had been previously shown to inhibit receptor binding, suggesting that MV could potentially escape such human antibodies albeit with decreased fitness.

## Results

### Measles Virus H can tolerate rather significant escape mutations, including N-linked glycosylation sites, in different immunodominant epitopes

The control virus for these experiments is MV-eGFP. This is a highly attenuated, laboratory adapted strain of vaccine Edmonston B ancestry expressing eGFP [34]. MV-eGFP propagates in Vero cells using the CD46 receptor but still has tropism for human SLAM and Nectin-4. All engineered and naturally selected mutations presented here, were within the H gene encoded by MV-eGFP and the mutated viruses were renamed as indicated.

**Figure 1:**
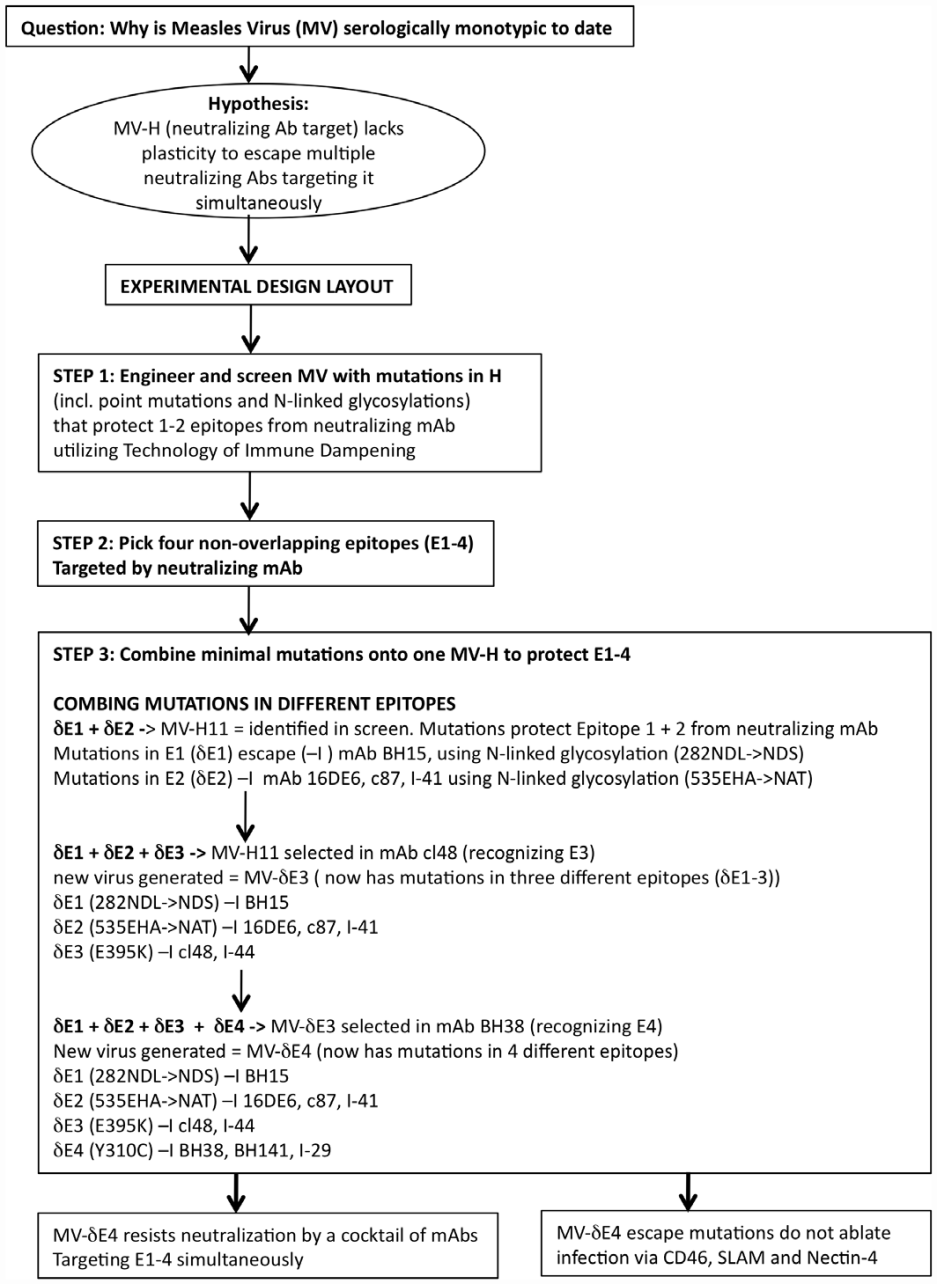
Scheme outlines the experimental design and results.

Our aim was to determine whether the monotypic nature of MV was due to a lack of structural plasticity of the H protein. A figure scheme (figure 1) outlines the experimental design and results. We used the technology of Immune Dampening [35], [36] to shield potential immunodominant epitopes from neutralizing monoclonal antibodies (mAbs). Epitopes were modified with rationally designed potential N-linked glycosylation sites (PNGS) and other amino acids substitutions. The rational design of potential escape mutations relied strongly on the analysis of the H crystal structure and previously published immunodominant epitopes and mAb escape mutations [8], [12], [13], [37], [38], [39], [40], [41], [42], [43], [44]. To permit viral replication, rationally designed mutations within known and putative epitopes were, for the most part, placed in flexible surface loops and residues that interacted with receptors were avoided.

The H protein was subjected to rounds of mutagenesis while being encoded in an expression plasmid. Following mutagenesis the bioactivity of H was monitored in vitro as the ability to induce cell-cell fusion of Vero cells when co-expressed with MV fusion (F) protein (table S1), Bioactive H mutants, encoding mutations in 1–2 potential epitopes, were cloned into MV-eGFP and a panel of viruses with mutated H proteins (MV-H#) were rescued. All MV-H# viruses grew to comparable titers as the control virus MV-eGFP, reaching 10^7^ TCID_50_/ml following three passages (table S1). Representative growth curves for four of the viruses discussed in this study are shown in figure S2.

Since we only modified one to two potential epitopes within H per MV-H# virus, we did not expect that these modifications would confer resistance to pooled MV-immune human sera compared to MV-eGFP in a plaque reduction neutralization test (PRNT) (table S1).

### Screen of MV-H mutants used to select four non-overlapping epitopes surrounding the top half of the H cuboidal head domain

We screened the panel of MV-H# viruses against a panel of mAbs in an *in vitro* neutralization assay (table S1). A plaque reduction neutralization test (PRNT) was initially performed to determine the neutralizing concentration of each mAb (concentration required to neutralize MV-eGFP by 100%) (data not shown). A neutralizing concentration of mAb was then used for all subsequent neutralization assays to screen MV-H mutants. The neutralizing assay was performed in a 96 well format. We incubated MV-H# in neutralizing concentrations of mAb or media (control) prior to infection. Two days following infection the number of infectious foci were quantified by counting the number of eGFP expressing syncytia per well. A virus escaped neutralization if the number of syncytia in the presence of mAb was the same as in the absence of mAb (control). A virus was deemed partially resistant when a mAb reduced infection by 35–50% compared to 100% reduction of the control virus.

We used escape mutations identified in this screen to cluster mAbs into four groups targeting four non-overlapping epitopes: E1–E4 (table S2). To test the hypothesis if MV-H had the plasticity to tolerate mutations that resisted neutralization by mAbs inhibiting receptor binding, we include epitopes targeted by mAbs I-29, I-41, 16DE6 and I-44. These mAb had previously been shown to inhibit binding between soluble H protein and receptors CD46 and SLAM in *in vitro* binding studies [45].

**Figure 2:**
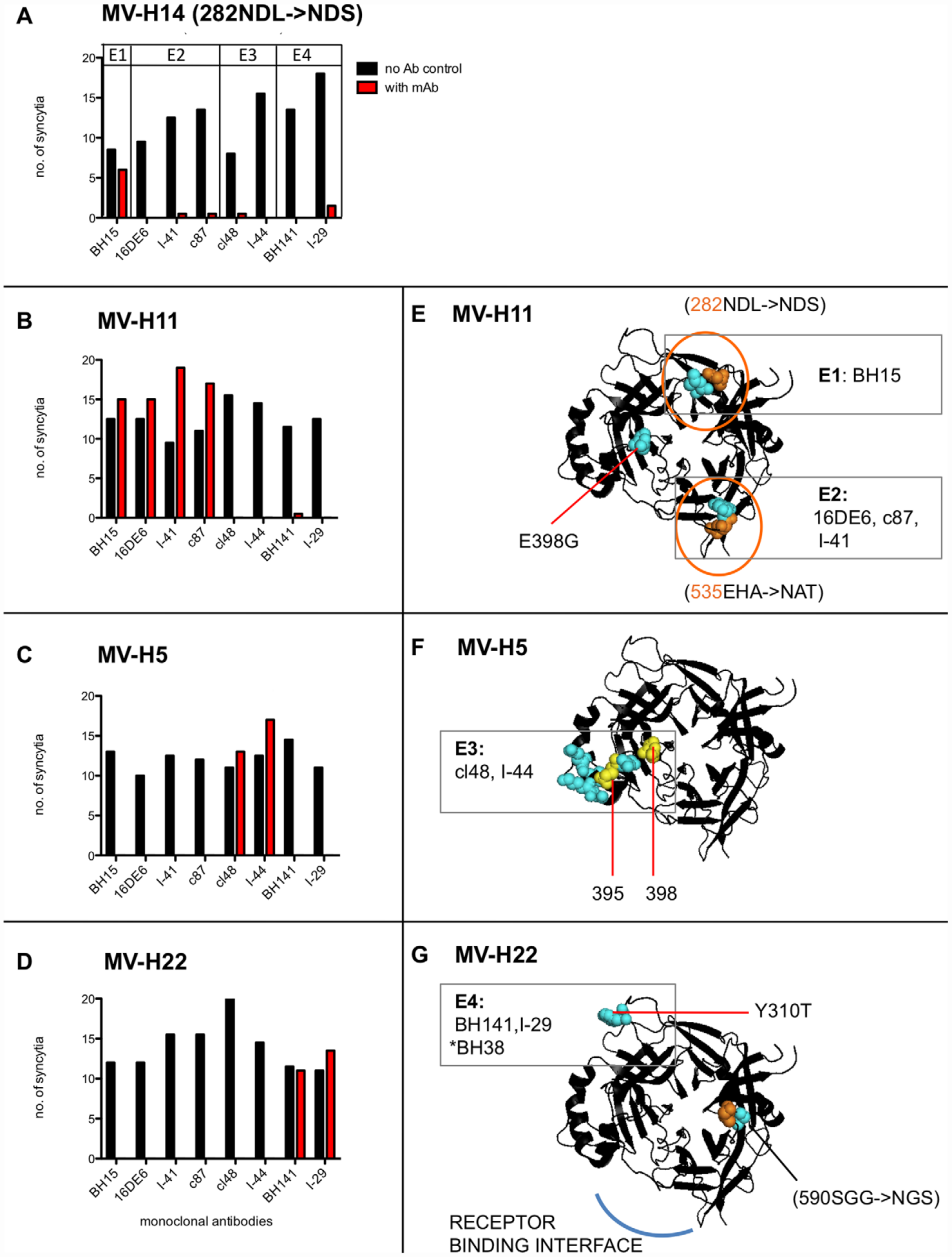
Rationally designed mutations delineate four different immunodominant epitopes of hemagglutinin protein recognized by neutralizing monoclonal antibodies. **A**) MV-H14, **B**) MV-H11, **C**) MV-H5 and **D**) MV-H22 were incubated in media (black bars) or neutralizing concentrations of BH15, 16DE6, I-41, c87, cl48, I-44, BH141 and I-29 (red bars) for 1.5 hours at 37°C. Infection in Vero cells was scored as the number of eGFP positive syncytia per well, 48 hrs post infection. Experiments were performed in a 96-well format in duplicate wells. Cartoon structures of the H cuboidal ectodomain (PDB 2ZB6) are shown as viewed downwards (Top View). They illustrates the escape mutations in **E**) MV-H11, **F**) MV-H5, **G**) MV-H22 and the mAbs they escape. The general location of epitope E1–4 is boxed and delineated by the location of the escape mutation(s) in each virus. All mutations are shown as spheres. Orange spheres highlight Asparagines (N) available for N-linked glycosylation within a PNGS. Orange circle illustrates a confirmed N-linked glycosylation. Yellow spheres in **F**) MV-H5, highlight mutated residues 395 and 398 for future reference. MV-H22 (**G**) does not escape *BH38, but previous studies localize it to E4 by virtue of a mutation in residue 310.

**Figure 3:**
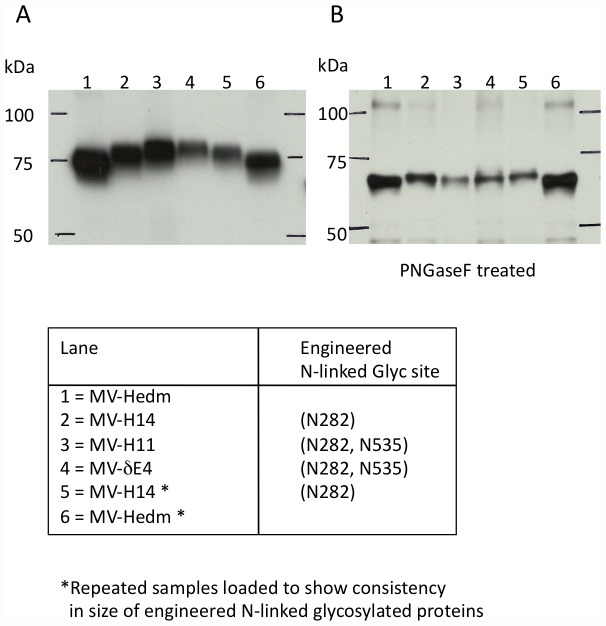
Glycosylation of engineered PNGS confirmed by western blotting of untreated and PNGase F treated viral H. **A**) MV-eGFP unmodified H encodes 5 PNGS. 4/5 PNGS have previously shown to be glycosylated (N168, N187, N200, N215). Therefore, MV-eGFP H (617 amino acids) migrates at 74 kDa. Upwards band shift is seen in MV-H14 (lane 2,5), which has one additional PNGS at residue 282 and a higher band shift is seen in MV-H11 (lane 3) and MV-δE4 (lane 5), both of which have two additional PNGS at residues 282 and 535 and migrate slower than H11. **B**) Downward band shift is seen in all the H proteins following the removal of N-linked glycans using by treating viral particles with PNGaseF.

We mapped E1–4 onto the crystal structure of the H cuboidal head ectodomain (2ZB6.PDB) (figure 2) based on the location of the escape mutations identified in the screen (table S1, table S2). E1–4 mapped to the top half of the head domain (figure 2 E–G), so we only show the top view of the head domain in our structural figures. E3 was protected by mutations in MV-H3-H5 and MV-H16 (table S1). These mutations all lie within or adjacent to the hemagglutinin-neutralizing epitope (HNE) (amino acids 380–400), which is recognized by hemagglutination-inhibiting, neutralizing and protective mAb and human antibodies in sera of some individuals [40], [42]. The HNE is also referred to as the noose epitope because of three cysteins: C381, C386, C394 that form a loop [42]. For simplicity we only mapped MV-H5 (figure 2,F). None of the rationally designed MV-H# mutants screened escaped BH38, but previous studies identified BH38 escape mutations as Y310D and L296I [8]. Since, residue 310 formed part of the E4 epitope, BH38 was grouped into E4 (figure 2D,G). N-linked glycosylation of rationally designed PNGS, protecting E1 (282NDL→NDS) and E2 (535EHA→NAT), was confirmed by western blotting of untreated and PNGaseF treated viral H proteins (figure 3). The PNGS at 590 SGG→NGS (figure 2D,G) was not glycosylated (data not shown) – this mutation was also not responsible for mAb resistance (MV-H20, table S1). Hence, only residue 310 forms part of the E4 epitope in MV-H22.

### Generation of a MV-H mutant escaping monoclonal antibodies targeting three different epitopes (MV-δE3)

**Figure 4:**
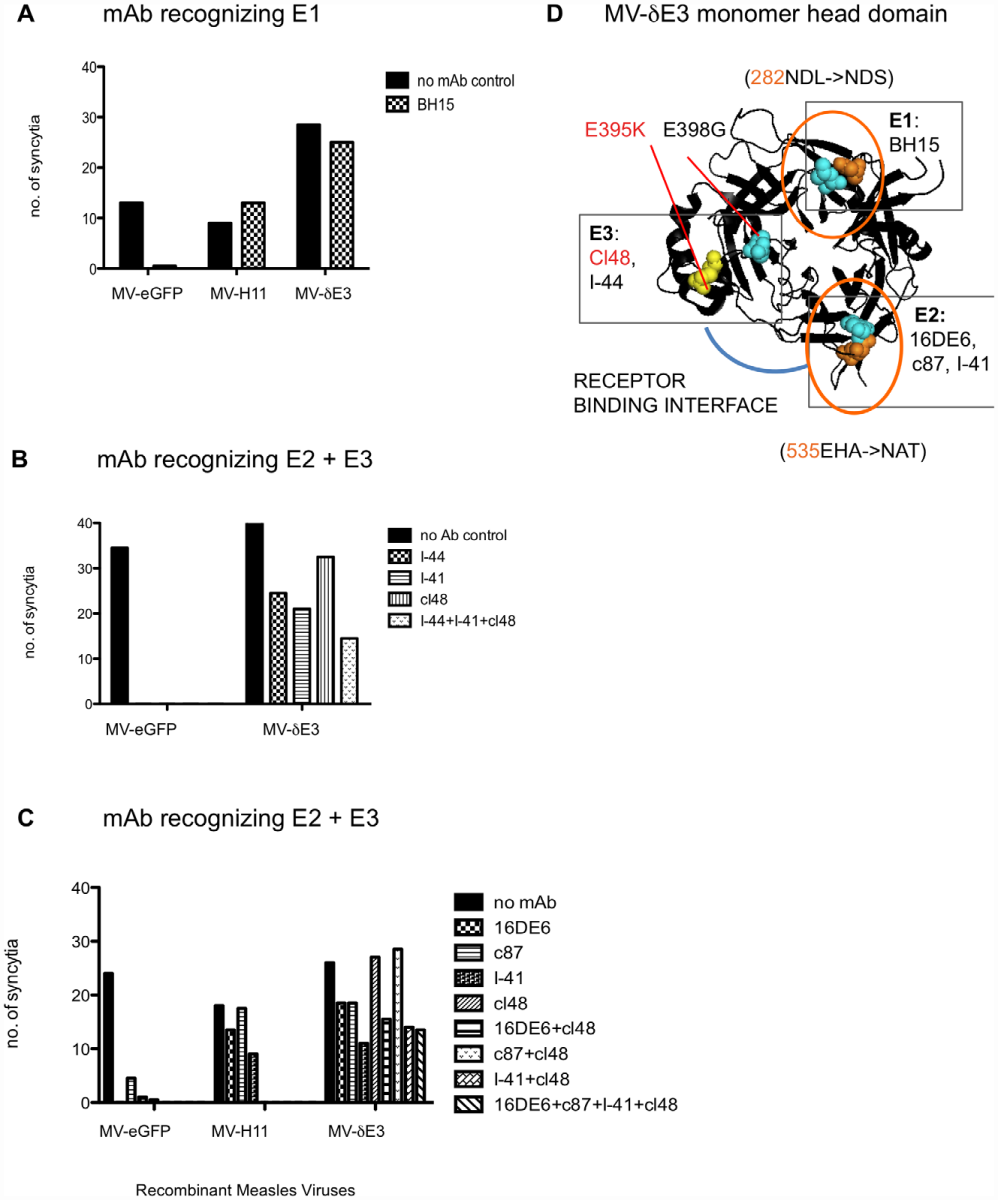
MV-δE3 resists neutralization by monoclonal antibodies targeting E1–3. The ability of MV-δE3 to escape monoclonal antibodies recognizing E1–3 was assessed in Neutralization Assays. MV-eGFP, MV-H11 and MV-δE3 were incubated in the absence (black solid bars) or presence (hatched bars) of mAbs prior to infection of Vero Cells. Infection was scored 48 hrs latter, as the number of eGFP positive syncytia per well. Experiments were performed in a 96-well format in duplicate wells. Viruses were challenged with mAbs targeting E1 (**A**) and E2 and E3 (**B,C**) in different combinations. **D**) Cartoon structure of MV-δE3 H cuboidal ectodomain illustrates all mutations as spheres. Orange spheres are Asparagines (N) available for N-linked glycosylation in PNGS 282NDL→NDS and 535EHA→NAT. Orange circles represent N-linked glycan shields. CL48 selected escape mutations E395K is represented by a yellow sphere. E1–3 are delineated by the location of the escape mutations and are boxed with the mAbs that recognize them.

Our aim was to generate a MV-H mutant with minimal escape mutations protecting E1–E4 from neutralizing monoclonal antibodies. MV-H11 already had two N-linked glycan shields at N282 and N535 shielding E1 and E2 (figure 2E). To add escape mutations that would protect E3 from mAbs, we naturally selected MV-H11 in neutralizing concentrations of mAb cl48 recognizing E3. The rational behind the artificial selection protocol was based on the fact that RNA viruses existed as a quasispecies population, due to the low fidelity of the RNA polymerase. MV mutation rate is of the order of ∼10^−5^ per nucleotide per replication cycle [22], [23], which equates to about one mutation per genome. Hence, in a quasispecies population of 10^6^ PFU, we anticipated that we would select at least one mAb escape mutation by incubating the virus in neutralizing concentrations of mAb, unless the escape mutation was deleterious to viral propagation. Following artificial selection of MV-H11 in cl48, we isolated a resistant clone with the escape mutation E395K and called the virus MV-δE3 (figure 4D). Our initial screen showed that MV-H3-H5 (table S1) completely or partially escaped cl48 and I-44. These viruses all had a mutation in residue 395 as well as 398. A single E398G mutation in MV-H11 and MV-H23 (table S1) did not protect E3, but may be complimentary in the context of E395K in MV-δE3.

MV-δE3 was either partially or completely resistant to mAb targeting E1–3 independently or in different combinations (figure 4 A–C). We particularly mixed mAbs targeting E2–3 in different combinations, as these epitopes were on the outskirts of the receptor-binding interface. Neutralizing mAb I-41, reduced MV-δE3 infection by 50%, indicating that the I-41 epitope was not completely protected in the context of mutations in MV-δE3. Artificial selection did not disrupt pre-existing resistance to monoclonal antibodies targeting E1 and E2 (Figure 4 A–C) and so we continued to use this technique to select escape mutations in E4.

### Generation of a MV-H mutant escaping monoclonal antibodies targeting four different epitopes (MV-δE4) simultaneously

**Figure 5:**
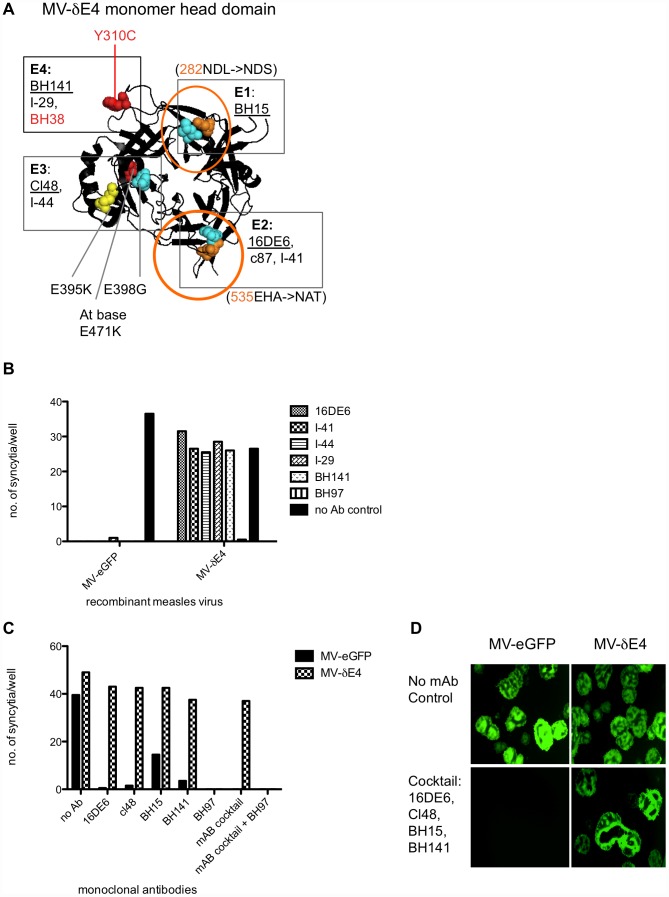
MV-δE4 evades neutralization by a cocktail of mAbs targeting E1–4 simultaneously. **A**) Cartoon structure of the MV-δE4 H cuboidal ectodomain (Top View) illustrates mutations as spheres. Orange spheres are Asparagine (N) residues available for N-linked glycosylation in PNGS 282NDL→NDS and 535EHA→NAT. Orange circles represent glycan shields. Yellow spheres highlight cl48 selected escape mutations E395K. Red spheres highlight BH38 (red) selected mutation Y310C and also E471K (which was present in ¼ BH38 resistant clones sequenced). Box highlights E1–4, delineated by the location of mutations escaping mAbs present in each box. Underlined are mAbs used in the cocktail mix in **C**) and **D**). **B)** MV-eGFP and MV-δE4 were incubated in the absence (black solid bar) and presence (hatched bars) of individual mAbs prior to infection of Vero cells. Infection was scored 48 hours latter by counting the number of eGFP expressing syncytia per well. BH97 was used as a positive control for MV-δE4 neutralization. **C**) MV-eGFP and MV-δE4 were then challenged with media alone (no mAb), a cocktail of mAbs targeting all four epitopes simultaneously: BH15 (E1), 16DE6 (E2), cl48, (E3) and BH141 (E4) with or without BH97 (control). **D**) Infection was visualized 48 hrs post infection by fluorescence microscopy at 4× magnification.

To protect the fourth and final epitope in this study, we select MV-δE3 in neutralizing concentration of BH38 recognizing E4. BH38 resistant clone, MV-δE4, encoded the escape mutation Y310C (figure 5A). This is the second time residue 310 has been implicated in the BH38 epitope. Previously, Y310D and L296I were identified as escape mutations in a BH38 escape MV variant [8]. Furthermore, Y310C in MV-δE4 provided resistance against other E4 targeting mAbs, BH141 and I-29 (figure 5B). Since we used our limited stock of BH38 during this artificial selection procedure, we did not include it in further neutralization assays to monitor resistance to E4. Instead, we used BH141 and I-29.

As planned, MV-δE4 encoded escape mutations in four non-overlapping epitopes (E1–E4). Escape mutations consisted of two glycosylated PNGS at N282 (E1) and N535 (E2) and point mutations E398G and E395K in (E3) and Y310C in (E4) (figure 5A). Next we wanted to determine if MV-δE4 would escape neutralization by a cocktail of monoclonal antibodies targeting the four epitopes simultaneously. We incubated MV-δE4 in a mixture of one mAb from each of the four groups: BH15 (E1), 16DE6 (E2), cl48 (E3), BH141 (E4). BH97 targets a fifth epitope, E5 (table S1), so we used BH97 as a positive control for MV neutralization. It also allowed us to crudely monitor if the accumulation of escape mutations in different epitopes inadvertently provided protection against BH97, as this would imply a structural change in the head domain following selection. MV-δE4 resisted neutralization by a cocktail of mAbs targeting E1–4 simultaneously, but was completely neutralized by control mAb BH97 (figure 5 C, D), for which it had no resistance.

### Escape mutations in MV-δE4 facilitate viral entry via CD46, SLAM and Nectin-4 but inhibit cell-cell fusion via SLAM and Nectin-4

Binding of soluble H to receptors CD46 and SLAM can be completely or partially inhibited by 16DE6, I-29, I-41 (E2, E4) and I-44 (E3) respectively [45]. Hence, we wanted to determine the effect of escape mutations in MV-δE4 on viral entry via human CD46, SLAM and Nectin-4. We kept in mind that rationally designed and naturally selected escape mutations were screened and selected solely on Vero cells expressing CD46 and that there was no screening criteria or selection pressure to select for escape mutations that facilitated entry via SLAM or Nectin-4.

**Figure 6:**
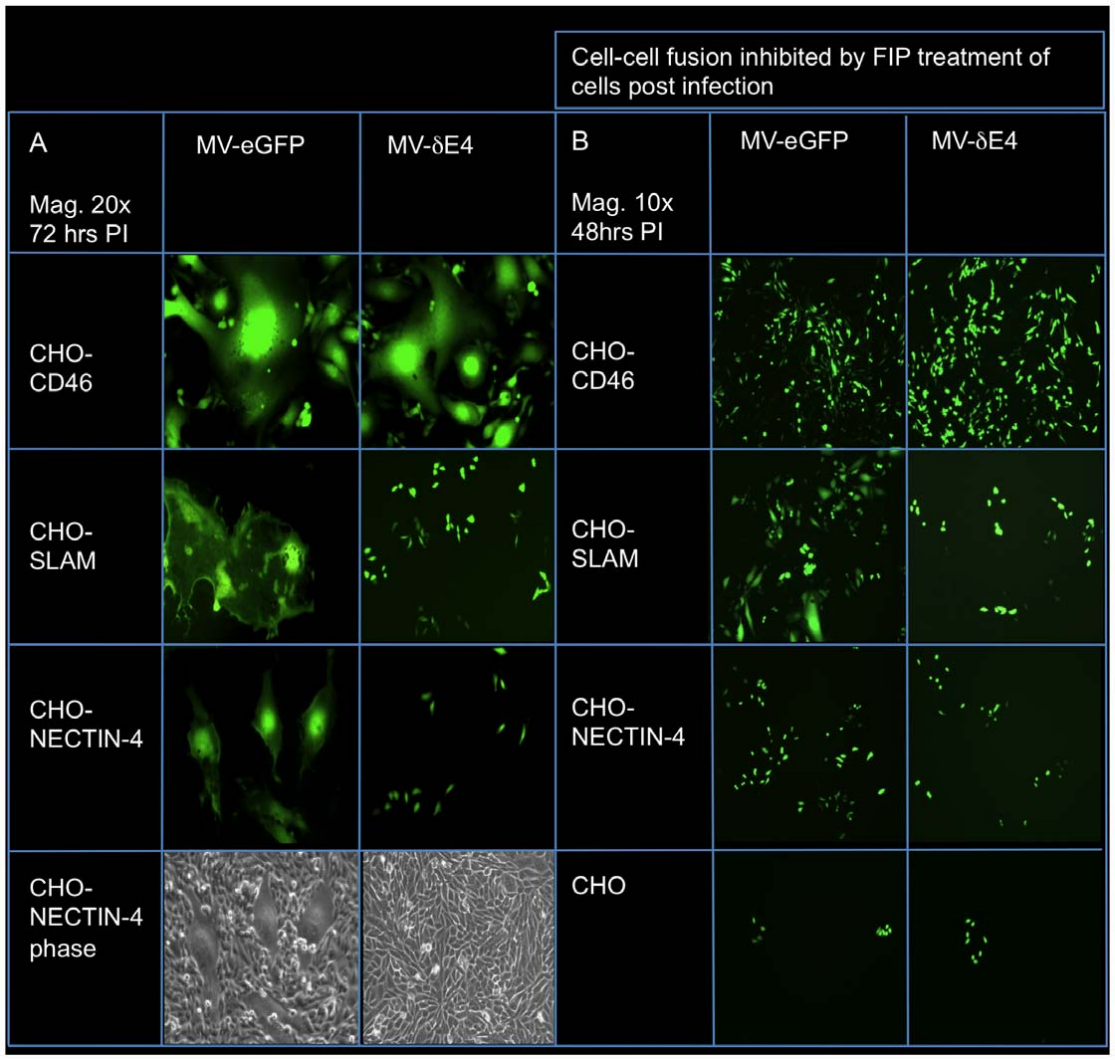
Escape mutations in MV-δE4 inhibit cell-cell fusion via Nectin-4 and SLAM receptors but not CD46. CHO cells or CHO cells stably expressing human CD46, SLAM or Nectin-4 were infected with MV-eGFP or MV-δE4. Twenty four-hours post infection (**A**) fresh media or (**B**) media containing FIP was added to the cells. (**A**) The extent of cell-cell fusion following infection was imaged 72 hrs post infection by fluorescent microscopy at 20× magnification. Mutations in MV-δE4 inhibited fusion via SLAM and Nectin-4 but not CD46. Infected CHO-Nectin-4 cells were imaged in phase to show the relative size of syncytia to single cells. (**B**) Cells were treated with FIP to inhibit fusion and the relative number of infected cells was imaged 48 hours post infection. Escape mutations in MV-δE4 decrease the level of infection via SLAM and Nectin-4 but not CD46. CHO cells can be infected at a very low level. MV entry into CHO cells occurs via an unidentified receptor.

CHO cells can be infected with MV at very low levels via an unidentified receptor but they do not support viral replication due to a cellular restriction. CHO cells expressing human CD46, SLAM or Nectin-4 are often used to study MV entry via these receptors. CHO, CHO-CD46, CHO-SLAM and CHO-Nectin-4 cells were infected with MV-eGFP and MV-δE4 in the absence or presence of fusion-inhibitory-peptide (FIP) to monitor relative cell-cell fusion and infection of individual cells respectively. FIP (Z-d-Phe-Phe-Gly ) is widely used for inhibition of paramyxovirus induced cell fusion. It acts by binding to the cell membrane [46] though the mechanism of action is unknown. MV-δE4 infected cells via all three receptors but the escape mutations ablated fusion in CHO-SALM, CHO-Nectin-4 but not CHO-CD46 cells. Loss of fusion also correlated to a decreased level of infection in these cells relative to cells infected with MV-eGFP (Figure 6).

### Monoclonal antibody MV escape mutants do not evade human serum neutralization

**Figure 7:**
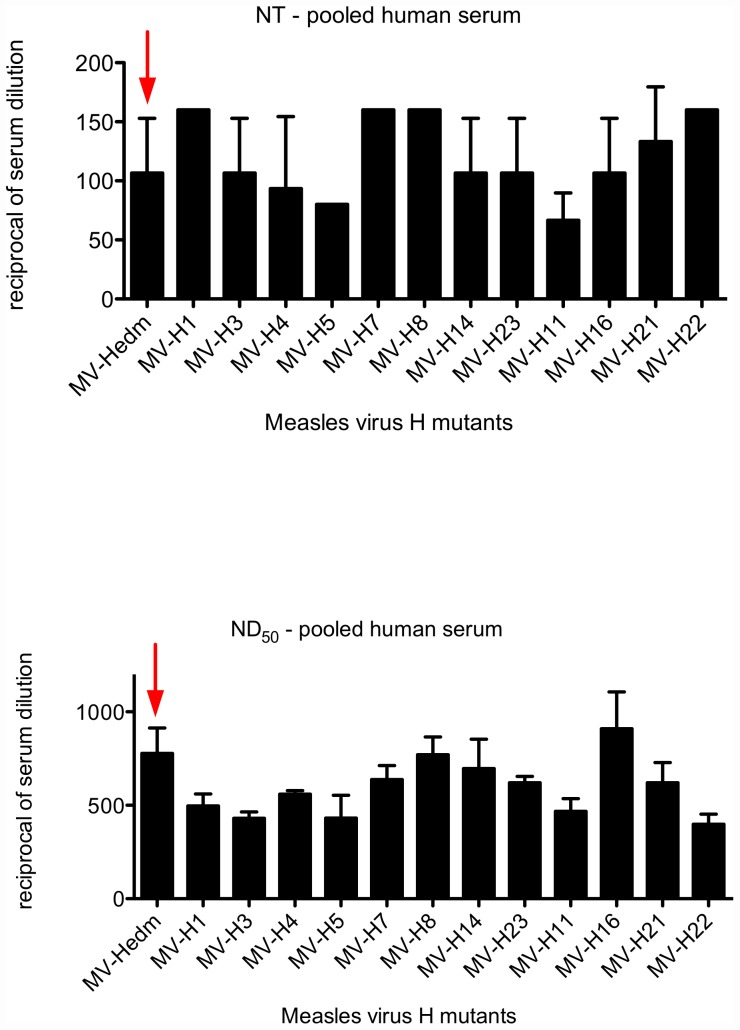
MV-H mutants with 1–2 epitopes eliminated/shielded do not escape neutralization by pooled human serum. Plaque Reduction Neutralization Test analyzed the susceptibility of MV-H mutants to neutralization by anti-MV antibodies present in pooled human serum. Viruses were incubated in 2-fold dilutions of serum for one hour at 37°C prior to infection of Vero cells. Plaques in the different dilutions were counted and compared with the virus-only control to calculate the 50% Neutralization Dose (ND_50_) using Karber's formula [79] and to determine the Neutralization Titer (NT, dilution of serum neutralizing 100% of the virus). We consider a MV-H mutant to be less sensitive to serum neutralization if it has a NT and ND50 that is >2 –fold lower than the NT and ND_50_ for the control virus MV-Hedm).

**Figure 8:**
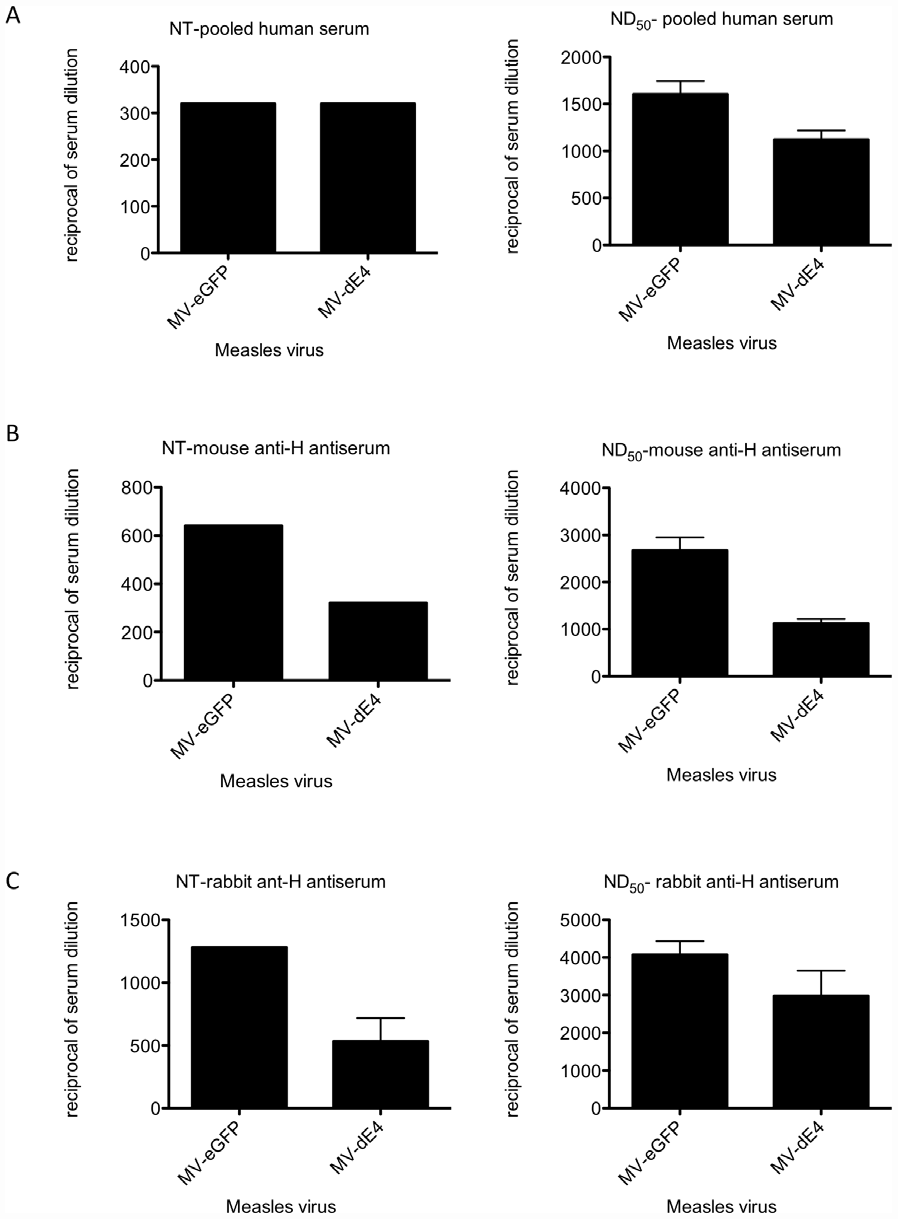
MV-HδE4 is neutralized by pooled human serum but is less sensitive to neutralization by anti-mouse and anti-rabbit anti-H antibodies. **A**) Plaque Reduction Neutralization Test analyzed the susceptibility of MV-H**δ**E4 to neutralization by anti-MV antibodies present in pooled human serum, **B**) anti-mouse anti-H antibodies present in Immunized mouse serum and **C**) Anti-rabbit anti-H antibodies present in immunized rabbit serum. Viruses were incubated in 2-fold dilutions of serum for one hour at 37°C prior to infection of Vero cells. Plaques in the different dilutions were counted and compared with the virus-only control to calculate the 50% Neutralization Dose (ND_50_) using Karber's formula [79] and to determine the Neutralization Titer (NT, dilution of serum neutralizing 100% of the virus). We consider a MV-H mutant to be less sensitive to serum neutralization if it has a NT and ND_50_ that is >2 –fold lower than the NT and ND_50_ for the control virus MV-Hedm).

To assess if the MV H mutants evade human serum neutralization we performed a fluorescence-based plaque reduction microneutralization test (PRNT). Prior to infection of Vero cells, MV H mutants were incubated for one hour at 37°C in serial dilutions of pooled human serum collected from ∼100–150 male donors at FDA-licensed commercial donor centers within the US. Two days post infection eGFP expressing syncytia at each serum dilution were counted. The percentage of infection at each serum dilution relative to infection in the absence of serum was used to calculate the 50% neutralizing dose (ND_50_, reciprocal of serum dilution that neutralized 50% of the virus) and the complete neutralizing titer (NT, reciprocal of serum dilution that neutralized 100% of the virus). Due to the fact that we often see a 2-fold difference in the ND_50_ and NT for MV and MV-H mutants between repeat PRNT experiments, we only consider a MV-H mutant to be less sensitive to serum neutralization if it has a NT and ND_50_ that Is consistently 2–fold or more lower than that for control virus MV-eGFP. Our analysis demonstrates that all MV-H mutants (figure 7) and MV-δE4 (figure 8) were as susceptible to human serum neutralization as MV-eGFP.

Human sera contains MV-neutralizing antibodies against H and F protein but the majority of the antibodies are directed against the H protein [47], [48]. To eliminate the contribution of anti-F antibodies to MV-δE4 neutralization by polyclonal serum, we performed the PRNT test with mouse and rabbit anti-H antisera generated following immunization with Adenovirus transducing only the H (nse) glycoprotein (figure 8). Both sera consistently had ∼2-fold lower ND_50_ and NT values for MV-δE4 than for MV-eGFP. Dominant epitopes recognized by mouse and rabbit antibodies may differ to dominant epitopes recognized by human antibodies. Since we targeted only epitopes recognized by monoclonal antibodies it would explain why the virus is less susceptible to mouse anti-H anti-sera and not human serum. Furthermore both the mAbs and the mouse anti-H antisera was generated by BALB/C mice.

## Discussion

Our aim is to better understand the factors restraining the measles virus to one serotype. In this study we probed the structural plasticity of the viral H protein, as it is the major target of neutralizing antibodies in sera of individuals protected by either vaccination or wild-type infection. We explored different escape mutations in immunodominant epitopes of the MV H glycoprotein and screened for mutations that allowed the virus to escape neutralization by mAbs (table S1). In particular, we focused on PNGS, which when glycosylated could shield neighboring residues recognized by mAbs. Escape mutations screened in table S1 did not result in a significant drop in viral titer. We then started combining escape mutations using a combination of rational design and artificial selection and generated a MV mutant resistant to a cocktail of mAbs targeting four different epitopes, two of which were protected by N-linked glycan shields (figure 5).

Epitopes E1–4 modified in MV-δE4, appear to overlap with epitopes recognized by human antibodies (Abs). This was demonstrated in competition ELISA studies where mAb competed with human Abs in serum of vaccinees or late convalescent, for binding to MV-H protein. mAbs that inhibited human Abs from binding to MV-H to the greatest degree were BH26 and mAbs that recognize the HNE (H380–400). BH26 was not explored here, but evidence suggests that BH26 binds to linear epitope H571–579 or H190–200 [8]. mAbs that recognized the HNE in our study were cl48 (recognizing E3) based on its E395K escape mutation (figure 5). mAbs that inhibited human Ab from binding to MV-H to a slightly lesser degree were BH15 (recognizing E1) and BH38 (recognizing E4) (figure 5) [40]. In a different study, 16DE6 and I-41 (recognizing E2) inhibited MV binding to human recombinant Fab fragments isolated from RNA derived from bone marrow or splenic lymphocytes from immune donors [41]. Despite this apparent overlap between E1–4 and epitopes targeted by human antibodies, MV-δE4 did not escape neutralization by pooled human serum in a plaque reduction neutralization test (PRNT). This was not surprising as we demonstrated that a single mAb targeting a non-protected epitope, such as BH97, would neutralize the virus completely. Hence, all the epitopes recognized by neutralizing human antibodies may need to carry escape mutations for the virus to escape neutralization from a polyclonal response in serum.

It has been proposed that MV exists as a single serotype because the receptor-binding interface, which is a target for mAbs, is highly unfavorable to mutation due to its functional importance [10], [12], [13]. Binding studies between soluble H protein and receptors CD46 and SLAM found that H protein binding to both receptors was completely blocked by I-29, I-41 and 16DE6 and 50% inhibited by I-44 [45]. MV-δE4 resisted neutralization by all these mAbs and escape mutations did not significantly effect viral propagation or syncytia formation via MV receptor CD46 expressed in Vero cells (Figure 5C–D). However, affinity for SLAM was reduced, as deduced from the loss of cell-cell fusion and a reduced level of infection in CHO cells expressing human SLAM (figure 6). This is not the first time mAb escape mutations have interfered with SLAM binding. Previously, Hu and colleges [38] also selected the MV LEC-WI strain on Vero cells (endogenously expressing CD46) in the presence of mAbs I-29, I-41, 16DE6 and I-44 (table S3) [38], [43]. Selected 16DE6 and I-41 escape mutations were in SLAM binding residues R533 and F552 [12], [13], respectively (table S3). Notably if MV was selected on cells expressing SLAM as the sole receptor instead of CD46, selection pressure may have selected for escape mutations that did not compromise infection via SLAM. If the SLAM binding interface was the ultimate neutralizing epitope, then one would anticipate that MV selected in the presence of human serum on Vero cells would be able to sacrifice SLAM binding to escape human Abs and acquire some degree of protection against neutralization. However our attempts at selecting MV in the presence of human serum from a vaccinated individual on Vero cells were futile (data not shown). Mutations rationally designed for our screen omitted residues involved in receptor binding, so to shield both the epitopes recognized by 16DE6 and 1–44 (as defined by [38], table S3) we placed a PNGS at residue 535 (figure S1b), whose glycosylation we confirmed (figure 3). Although this rationally designed modification did decrease the level of infection via SLAM – as it was still in the SLAM-binding interface - it did not ablate it. Placing the PNGS further away from SLAM binding residues may successfully shield 16DE6 and/or I-44 epitopes without affecting infection via SLAM. The Asn available for glycosylation would need to be in spatially proximity to the epitope, so that the N-linked glycan can mask it. We have demonstrated this in the mutant MV-H16 (defined in table S1). Here a glycosylation at residue 403 was able to protect MV from cl48, whose epitope we localized to E3 (HNE) (residue 395) (figure 4, figure S1a).

An analysis of synonymous and non-synonymous mutations present in H from all MV genotypes was used to determine which residues were subjected to selection pressure from the human humoral immune system [49]. Residues under positive selection included 302, 451, 481, 560, 562, and 575, if Subacute sclerosing panencephalitis (SSPE) strains are excluded from the analysis. Residues involved in SLAM and Nectin-4 binding were not under selection, but residues 451 and 481, which interact with CD46 were. Notably, oncolytic measles viruses retargeted by a polypeptide-ligand displayed on the H C-terminus have SLAM and CD46 binding sites on H ablated, which include residue 481 [50]. Retargeted oncolytic MVs were as susceptible to pooled human serum neutralization as non-retargeted MV-eGFP, even though they encoded mutations ablating receptor binding; escaped mAbs targeting the receptor binding surface and attached to receptors via a separate domain linked to H C-terminus (paper in submission – Lech et. al.). This suggests that even if wild-type MV could escape antibodies targeting the receptor binding surface it would still be neutralized. All sites identified by [49] that experience positive selection differ from monoclonal antibody epitopes analyzed in this study and should be explored in more detail in the future.

In summary, restraints on MV that confine it to one serotype may be more complex than its inability to tolerate multiple escape mutations within and around the receptor binding interface, when one takes the following into account. 1) MV has some degree of flexibility in the residues it can mutate to escape neutralization from mAbs that interfere with receptor binding. Hence, mutations in receptor binding residues could potentially be avoided (table S3). 2) Additional N-linked glycosylations are tolerated and can be used to shield neighboring epitopes (figure S1). 3) Attempts at evolving, a CD46 tropic, laboratory adapted MV in human serum on Vero cells was unsuccessful, even though SLAM and Nectin-4 binding sites could be sacrificed (unpublished data). 4) Neutralizing antibodies have also been mapped to regions other than the receptor-binding interface [8], [38], which may be functionally critical to the hetero-oligomer fusion complex. 5) Escape mutations may need to be present in all the epitopes recognized by neutralizing human antibodies for the virus to escape neutralization.

We did not assess the fitness of MV-H mutants in vivo. We did analyze the fitness of some of the MV-H mutants using in vitro growth curves and did not see a significant difference in their growth kinetics (figure S2). Notably, even a marginal decrease in fitness in vivo could be a barrier to serotypic diversity when a virus needs to accumulate mutations in multiple epitopes, with each additional mutation being at a cost to fitness. Escape mutations may also revert in the absence of selecting pressure after transmission to a new host [51]. Or, new compensatory mutations that correct a loss of fitness due to escape mutations may be selected for by the virus [52].

Two factors that drive the rapid genetic change for many viruses are mutation rates and recombination. MV depends primarily on its mutation rate, as recombinations have been rarely reported in paramyxoviruses [53], [54]. This may impede its rate of evolution. One factor limiting recombination may be the rule of 6, which states that the genome length must be evenly divisible by 6 for the generation of viable progeny. The nucleocapsid protein, which binds hexamers of RNA, is thought to monitor and maintain hexamer genome length [55]. Recombination resulting in deletions or insertions that violate the rule of 6 would be deleterious [54], [56], [57]. The mutation rate of MV is similar to other RNA viruses [22] and has contributed to the evolution of 23 genotypes. Even though, serum samples from vaccinees neutralize viruses from all the genotypes, there is a substantial difference in neutralization titers, even amongst strains from the same genotype [6], [58]. Contributing factors may include the accumulation of mutations in different strains driven by selection pressure [49] and differences in the quality and quantity of anti-measles antibodies in individuals [6].

An important question is whether in the long run the serologically monotypic measles virus will remain a single serotype or whether this may change in the future. After vaccination antibody titers are usually lower than after wild-type infection [6], [59], [60], [61], [62]. Hence, there is a concern that vaccination may result in more opportunities for virus selection by humoral immune pressure, especially in individuals with low or waning antibody titers. Theoretically this could be possible with time, because today most of the world's immunity against measles is vaccine induced by genotype A strains [5]. Prior to wide spread introduction of vaccination, MV evolved under the selection pressure of an immune response to multiple genetically distinct wild-type MVs. Thus MV circulating strains were not always under the same immune pressure like they are today [5], [63], [64]. On the other hand, while antibodies alone can protect against measles, the T-cell response alone can too, as seen in individuals with antibody deficiencies [65]. Hence, the combination of T and B cell responses may also severely limit the serological evolution to other serotypes [66], [67], [68], [69]. Notably, as vaccine coverage increases the viruses regress, hence the opportunities for the virus to develop under immune pressure may become less.

We conclude, that in the event MV is driven by immune pressure to modify the epitopes explored in this study, it has some flexibility in the residues that can be mutated to resist neutralization. Additional N-linked glycan shields on H are tolerated in at least three epitopes analyzed and they protect a greater surface area i.e. can shield epitopes that are within spatial proximity to the PNGS. To better understand the evolutionary restraints imposed on MV, further studies are warranted to continue shielding other epitopes in MV-δE4 and monitor their effect on virus propagation, fusion and the formation of the hetero-oligomeric fusion complex. This is the first time demonstrating that rationally designed Measles viruses can be derived by molecular means that are capable of resisting multiple neutralizing antibodies.

## Materials and Methods

### Cell Culture

Vero African green monkey kidney cells and Chinese hamster ovary (CHO) cells were grown in DMEM with 5% FBS. CHO-CD46 cells [70] stably expressing human CD46 were maintained in DMEM, 10% FBS, 1 mg of G418/ml. CHO-SLAM cells [71] stably expressing human SLAM were grown in RPMI 1640, 10% FBS, 0.5 mg/ml of G418. CHO-Nectin-4 cells (kind gift from Marc Lopez) [72] stably expressing human Nectin-4 were maintained in RPMI 1640, 10% FCS, 0.5 mg/ml of G418, 0.1 mM MEM non-essential amino acids solution. All cell culture media was supplemented with 1% penicillin and streptomycin and cells were grown at 37°C in a humidified atmosphere of 5% CO_2_.

### Site-Directed Mutagenesis of rationally designed mutations

Mutations in H were performed in pCG-H (measles H glycoprotein (Nse strain) expression construct) using QuikChange® Site-Directed Mutagenesis Kit (Stratagene) as per manufacture's directions (Publication Number: 200518-12). Overlapping oligonucleotides were designed to introduce mutations in the H gene using pfuTurbo DNA polymerase (Stratagene). Following Dpn1 digestion of parental plasmid DNA, the synthetic DNAs were transfected into DH5α competent cells (Invitrogen). DNA sequence analysis was used to confirm the intended mutations and to ensure that the plasmids were free of unwanted second-site mutations. Each H mutant was designated a number (#), i.e. pCG-H5, H14, H11, H22. Potential N-linked glycosylation sites were generated by site-directed mutagenesis to generate a N-linked glycosylation sequon Asn-X-Ser/Thr. Where X is any amino acid except a proline [73]. Exhaustive determination of the glycosylation state of each mutated H protein was not performed in this study. However, previous work performed using similar mutations with the gp160 of HIV and the HA of influenza demonstrated that the mutations designed to introduce additional glycosylations results in the intended post-translational modifications [36], [74].

### Measles Fusion Assay

After the mutant H genes were confirmed by sequencing, the mutations were tested for bioactivity using a cell fusion assay. 6-well plates were seeded with 4 ml of Vero cells (3×10^6^) in Dulbecco's Modified Eagle's Medium (DMEM, Lonza) supplemented with 5% Fetal Bovine Serum (FBS, Lonza) 24 hrs in advance of the assay. The plates were incubated at 37°C in a humidified environment containing 5% CO_2_. The next day, the media was removed from the cells and replaced with 1 mL fresh, pre-warmed DMEM with 0% FBS. 2 µg of a plasmid encoding the measles fusion gene (F) was mixed with 2 µg of WT or mutant H DNA in a polystyrene tube and pre-warmed DMEM-0% was added to a total volume of 50 µl. 7 µl of FuGene (Roche) was directly added to the DNA mixture, the tubes were vortexed, and incubated at room temperature for 15 min. The DNA-FuGene mixture was added to each well dropwise, and mixed by gently rocking the plate side to side. The plate was incubated at 37°C for 30 min, then rocked again and incubated at 37°C for 5 hrs. 2 mL of DMEM- 5% was added to each well, rocked to mix, and returned to 37°C to incubate. Plates were examined under a phase-contrast microscope to observe cell fusion over the next three days. Fused foci of cells (syncytia) were scored on a three-point scale (+, +/−, and −).

### Plasmids, virus rescue and titration

To generate the full-length anti-genomic MV plasmids, in which the H glycoprotein was replaced by H#, p+MVeGFP [34], and the pCG−H# expression constructs were digested with *PacI* and *SpeI*, and H(Nse strain) was exchanged with the H# fragments to create the plasmids p+MVeGFP-H# (p+MV-H#). Correct assembly of the constructs was verified by dideoxy-sequencing. MV rescue system is well established [75]. Briefly, 293-3-46 cells were transfected with p+MVeGFP-H# or p+MVeGFP and nucleoprotein (N) and polymerase (L) proteins expression plasmids [76] using Calcium Phosphate Transfection Method. Seventeen hours post transfection, media was replaced with fresh media and the cells were heat-shocked at 42°C for 2.5 hrs. Seventy-two hours after transfection the 293-3-46 cells were overlayed onto Vero cells. Twenty-four hours latter eGFP expressing syncytia started appearing. Individual syncytia (clones) were picked and used to infect new Vero cells to amplify the virus. The virus was further amplified with two serial passages by infecting Vero cells at moi of 0.02 at 37°C. When 90% of the cells were incorporated into syncytia the cells were harvested by scrapping them off the plate in 1–2 mls of Opti-MEM (Invitrogen). The cell/virus suspension was subjected to three freeze-thaw cycles, centrifuged to clarify viral supernatant, aliquoted and stored at −80°C. Titers were determined by 50% tissue culture infective dose (TCID_50_) on Vero cells.

### Multi-step growth curve

Vero Cells were infected at a moi of 0.02 with MV-Hedm, MV-H5, MV-H11 and MV-HδE3. Eight hours post infection the media was replaced with fresh media. Cells were harvested every 12 hours and cell associated virons were released by lysing the cells by three freeze/thaw cycles. Lysates were centrifuged to clarify viral supernatant, aliquoted and stored at −80°C. Titers were determined by 50% tissue culture infective dose (TCID_50_) on Vero cells.

### Establishing a Neutralizing concentration of mAb

The ability of mAbs to inhibit MV-eGFP infection was assayed by the plaque reduction neutralization test (PRNT) in a 96 well format. mAbs were serially diluted in Opti-MEM (Invitrogen) and 50 uls was mixed with equal volume of MV-eGFP diluted to 4000 TCID_50_/ml (or 20–30 syncytia per well). The mixture was incubated for 1.5 hrs at 37°C before the addition of Vero cells (10,000 cells/well in 50 µl DMEM 5% FBS). Forty-eight hours post infection the number of eGFP expressing syncytia/well, were counted using a fluorescent microscope. The neutralizing concentration of mAb was the endpoint concentration of mAb required to reduce the number of MV-eGFP syncytia by 100%. Neutralization concentrations established for each mAb were as follows: 16DE6 (1.5 µl ascites/well); I-41 (1 µl ascites/well); I-44 (1.5 µl ascites/well); I-29 (6 µl ascites/well); BH141 (5 µl ascites/well); BH97 (2.5 µg/well); BH15 (3 µg/well) BH30 (0.8 µg/well); cl48 (1 µg/well); c87 (50 µl hybridoma supernatant).

### Neutralization Assay

Neutralization assay was performed in a 96 well format. Each sample was duplicated and the experiment was repeated 2 to 5 times depending on the mAb. Neutralizing concentration of mAb in 50 µl Opti-MEM or Opti-MEM alone (control) was mixed with equal volume of MV-eGFP or MV-H mutant viruses diluted to 4000 TCID_50_/ml (or 20–30 syncytia per well). In the case of mAb c87, 50 µl of hybridoma supernatant or 50 µl media was mixed with equal volume of virus. The mixture was incubated for 1.5 hrs at 37°C before the addition of Vero cells (10,000 cells/well in 50 µl DMEM 5% FBS). Forty-eight hours post infection the number of eGFP expressing syncytia/well, were counted using a fluorescent microscope.

### Artificial Selection of antibody escape mutations

Vero Cells were seeded into six-well plates at a density of 2.5×10^5^ cells/well and left to adhere overnight. Prior to infection, MV-H11 (1×10^6^ PFU) was incubated at 37°C for 2 hours in with 300 µg/ml cl48. MV-δE3 was incubated with 80 µg/ml BH38. The virus/antibody mixture was split between 6 wells of Vero cells. Viral replication was monitored for 72–96 hours. Virus from wells that showed the greatest degree of propagation was harvested and challenged again with antibody. This was repeated twice before syncytia were picked and amplified in 6 well plates in the presence of 40 µg cl48 or 10 µg BH38/well. Viral H genes were amplified by polymerase chain reaction (PCR) from individual clones and analyzed by dideoxy-sequencing to identify the escape mutation. Escape mutant propagation and titrations was performed as described earlier.

### N-linked deglycosylation and Immunoblot Analysis

Viruses (2×10^5^ PFU) were incubated with and without PNGase F for 1 hr at 37°C as per manufacturers protocol (New England Biolabs, P0705S), but without the initial denaturing step. SDS loading buffer was then added to the samples, boiled for 5 min at 95°C and separated on a 7.5% Tris-glycine SDS-polyacrylamide gel. Proteins were blotted to nitrocellulose membrane, immunoblotted with primary antibody: anti-rabbit anti- MV H protein (1∶6000 dilution) [77] and secondary antibody: HRP-conjugated goat anti-rabbit (1∶5000 dilution).

### Structural Modeling

The crystal structure of measles virus hemagglutinin protein PDB ID: 2ZB6 was analyzed and manipulated using Educational-use-only PyMOL software.

### Monoclonal Antibodies

Monoclonal antibodies used in this study were kind gifts from Drs. Claude Muller, Laboratoire National de Sante, Luxemburg (BH15, BH97, BH30, BH38, BH15), Erling Norrby and Mariethe Ehnlund, Karolinska Institute, Sweden (16DE6, I-41, I-44, I-29), Denis Gerlier, Inserm, France (c87), Mark Federspiel, Mayo Clinic, USA (purified cl48) but originally from T. Fabian Wild.

### Infection of CHO cells

CHO, CHO-CD46, CHO-Nectin-4 and CHO-SLAM cells were plated in a 96 well format and infected in triplicate. On the day of infection, media was removed from the cells and 50 µl of virus was added in Opti-MEM (Invitrogen). Cells were infected with recombinant measles viruses at a moi of 1 (moi calculated based on TCID_50_/ml as titered on Vero cells). Two hours post infection, 100 µl of media (cell type dependent) was added to the cells. Seventeen hours latter the media was replaced with fresh median or media with 200 µM fusion inhibitory peptide (FIP) (Bachem). Fluorescence microscopy was performed Four-eight and 72 hours post infection.

### Production of recombinant Adenoviral vector transducing MV-H glycoprotein

The H(nse) gene was cloned into pHCMV5R [78] using Not1 and Xba1 restriction sites. pHCMVR5 encoding Hnse gene was then digested with restriction enzymes I-Ceu1 and P1-Sce1 and cloned into pAdHM48 into the E1 position [78]. pAdHM48 with Hnse in position E1 was linearized with Pac1, purified by phenol-chloroform extraction and transfected into 293 producer cells using the Polyfect transfection kit (QIAGEN). After well-developed plaques formed, the cells were harvested and lysed by three freeze/thaw cycles. This inoculum was then added to 293 cells and the cycle was repeated 3 times to amplify the virus. The recombinant Adenoviral vector transducing H was propagated in 293 cells, and purified by centrifugation through a CsCl gradient. The vector stocks were then dialyzed against a 10% glycerol-PBS and frozen at −80°C. The adenoviral particle number was determined by measuring the optical density at 260 nm.

### Immunizations

Rabbit anti-H antisera was generated by immunizing a female NZW rabbit with Ad-H. Immunization was performed by intramuscular administration of 5×10^10^ virus particles in 600 µl (300 µl to each quadriceps) and intravenous administration of 5×10^10^ virus particles in 300 µl by ear vein. The rabbit received three inoculations (initial inoculations and 2 boosts) at three-week intervals. Mouse anti-H antisera was generated by immunizing BALB/C mice with Ad-H. Immunization was performed by intramuscular administration of 5×10^9^ virus particles in 50 µl (25 µl to each quadricep) and intravenous administration of 5×10^9^ virus particles in 100 µl by tail vein injection. Mice received three inoculations (initial inoculations and 2 boosts) at three-week intervals. Anti-H antibody titers in mouse and rabbit serum were established using a plaque reduction neutralization test.

### Fluorescence-based plaque reduction microneutralization Test

Pooled human serum (Human AB serum – sterile filtered, Valley Biomedical, product number HS1017, lot number C80553), mouse anti-H antisera and rabbit anti-H antisera, was heat-inactivated and diluted in 2-fold serial dilutions in Opti-MEM (Gibco, Invitrogen). 50 µl of each serum dilution was added to wells in a 96 well plate in triplicate. Control wells had 50 µl of Opti-MEM with no serum. MV recombinants were diluted in Opti-MEM to 4000 TCID_50_/ml (to give 20–30 syncytia/well). 50 µl of virus was added to wells containing serum dilutions or Opti-MEM. The virus/serum mixture was incubated for 1 hour at 37°C before the addition of Vero-His cells in DMEM, 5%FBS (10,000 cells/well). Plates were incubated for 44 hours at 37°C. Infection at each serum dilution was scored by counting the number of eGFP expressing foci per well using a fluorescent microscope. The number of eGFP expressing foci at each serum dilution compared to infection in the absence of serum was used to calculate the Neutralization Dose (ND50) using Karber's formula [79] and to determine the Neutralization Titer (NT, dilution of serum neutralizing 100% of the virus).

## Supporting Information

Figure S1 **N-linked glycosylation shields neighboring epitopes.** Cartoon structure of H monomer is shown in dark grey (top view). β-strands involved in binding to CD46, SLAM and Nectin-4 are colored in lime-green. **A**) MV-H16 has a rationally designed and glycosylated (orange circle) N-linked glycosylation site at 403 KDN→NDT. K403N available for glycosylation is shown as orange sphere and N405T is shown as a blue sphere. The glycosylation shields residue 395 (cyan) identified as the epitope for cl48 by virtue of the escape mutations E395K within the HNE (purple, spanning residues 380–400). **B**) Rationally designed and glycosylated (orange circle) N-linked glycosylation site 535EHA→NAT inhibits complete neutralization by I-41 and 16DE6. Alternative escape mutation for 16DE6 and I-41 are F552V (red sphere) and S532F or R533G (cyan spheres). Residue F552 and R533 (bold, underlined) are both involved in SLAM binding [13]. The HNE (380–400) is labeled to provide orientation.
(TIF)

Figure S2 **Multi-step growth curve analysis of MV-Hedm (black) and MV-Hmutants:** MV-H5 (orange), MV-H11 (blue) and MV-H**δ**E3 (black). Vero cells were infected with moi of 0.02 PFU/cell. Cell associated virons were harvested every 12 hours after incubation at 37°C.
(TIF)

Table S1 **MV-H# viruses with mutations in potential immunodominant epitopes screened to identify monoclonal escape mutations.**
(DOCX)

Table S2 **Mutations in MV-H# escape mutants delineate four different epitopes targeted by monoclonal antibodies.**
(DOCX)

Table S3 **Escape mutations against monoclonal antibodies that interfere with receptor binding are not confined to a single residue and can include a N-linked glycosylation site.**
(DOCX)
