# Supplementary figures and images for: Epitope Dampening Monotypic Measles Virus Hemagglutinin Glycoprotein Results in Resistance to Cocktail of Monoclonal Antibodies
Source: PLoS One. 2013 Jan 3;8(1):e52306. doi: 10.1371/journal.pone.0052306 (PMC3536790; doi:10.1371/journal.pone.0052306)

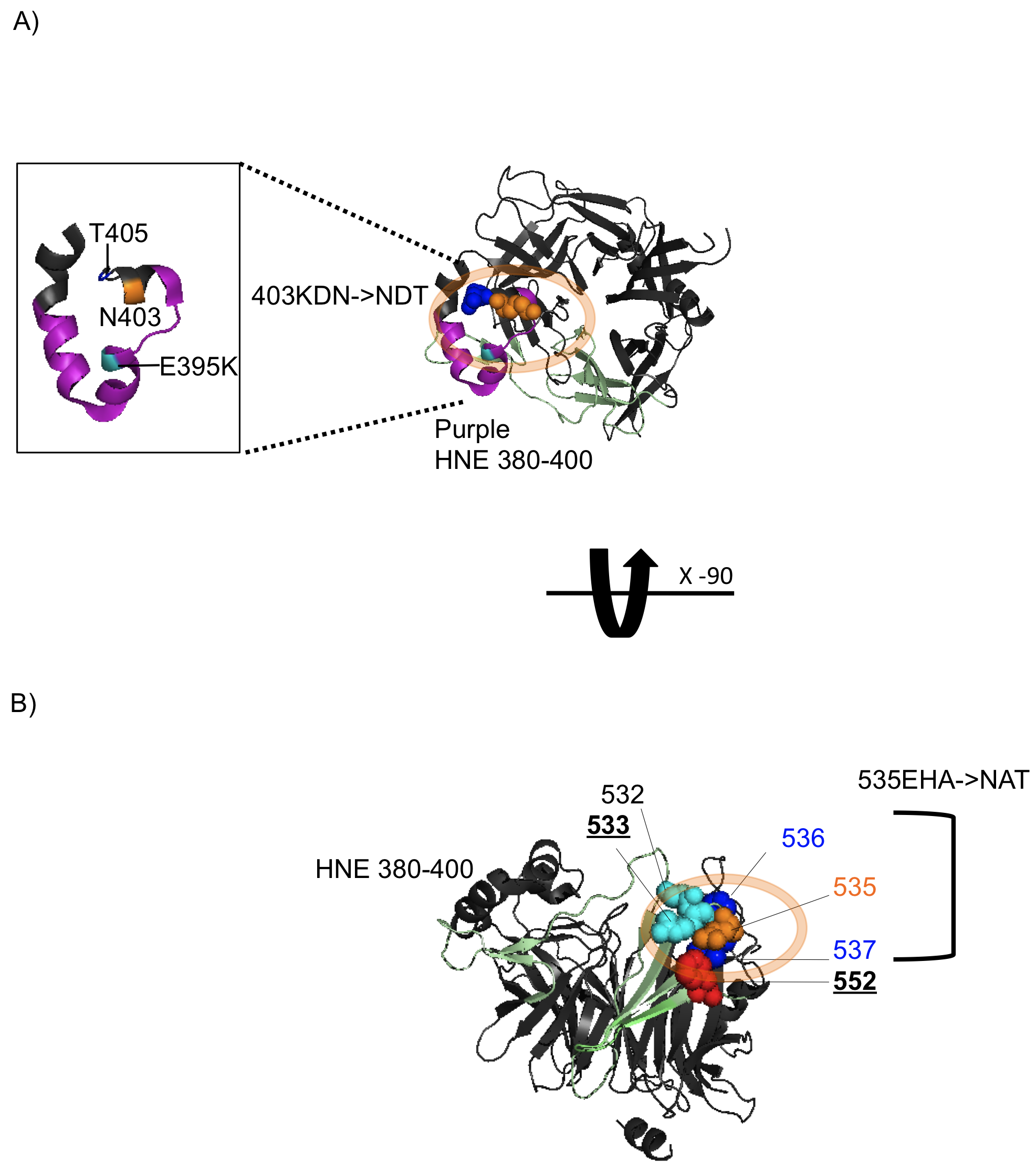

Supplement: Figure S1 — N-linked glycosylation shields neighboring epitopes. Cartoon structure of H monomer is shown in dark grey (top view). β-strands involved in binding to CD46, SLAM and Nectin-4 are colored in lime-green. A) MV-H16 has a rationally designed and glycosylated (orange circle) N-linked glycosylation site at 403 KDN→NDT. K403N available for glycosylation is shown as orange sphere and N405T is shown as a blue sphere. The glycosylation shields residue 395 (cyan) identified as the epitope for cl48 by virtue of the escape mutations E395K within the HNE (purple, spanning residues 380–400). B) Rationally designed and glycosylated (orange circle) N-linked glycosylation site 535EHA→NAT inhibits complete neutralization by I-41 and 16DE6. Alternative escape mutation for 16DE6 and I-41 are F552V (red sphere) and S532F or R533G (cyan spheres). Residue F552 and R533 (bold, underlined) are both involved in SLAM binding [13]. The HNE (380–400) is labeled to provide orientation. (TIF) [file pone.0052306.s001.tif]

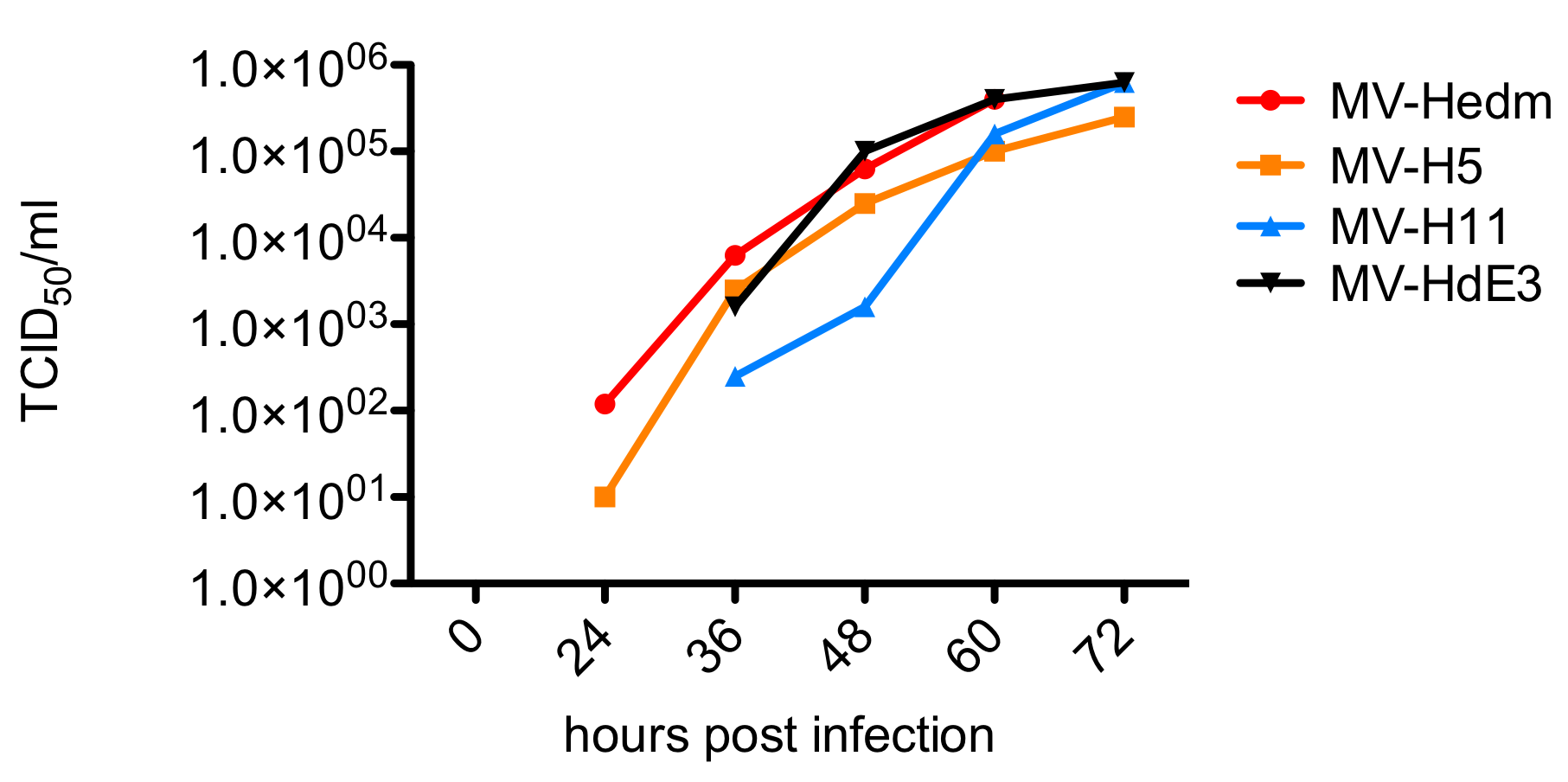

Supplement: Figure S2 — Multi-step growth curve analysis of MV-Hedm (black) and MV-Hmutants: MV-H5 (orange), MV-H11 (blue) and MV-HδE3 (black). Vero cells were infected with moi of 0.02 PFU/cell. Cell associated virons were harvested every 12 hours after incubation at 37°C. (TIF) [file pone.0052306.s002.tif]
