# Supplementary material for: Epitope Dampening Monotypic Measles Virus Hemagglutinin Glycoprotein Results in Resistance to Cocktail of Monoclonal Antibodies
Source: PLoS One. 2013 Jan 3;8(1):e52306. doi: 10.1371/journal.pone.0052306 (PMC3536790; doi:10.1371/journal.pone.0052306)
Supplement: Table S1 — MV-H# viruses with mutations in potential immunodominant epitopes screened to identify monoclonal escape mutations. (DOCX) [file pone.0052306.s003.docx]

| **MV-H#** | **Rationally designed mutations** | | **Fusion** | **TCID_50_/ml** | **R. human serum *** | **R. to mAb** | **Epitopes** |
| --- | --- | --- | --- | --- | --- | --- | --- |
| **Amino Acid Substitutions** | | | | | | | |
| H3 | E395D, N396Q, E398D | | Yes | 10^7^ | No | Cl48, I-44 | E3 |
| H4 | Q391G, E395A, N396G, E398G, | | Yes | 10^7^ | No | Cl48, I-44 | E3 |
|  | A400V | |  |  |  |  |  |
| H5 | Q383N,A385G,K387R,G388A,E395D, | | Yes | 10^7^ | No | Cl48, I-44 | E3 |
|  | N396G, E398D | |  |  |  |  |  |
| H7 | R547G | | Yes | 10^7^ | No | - |  |
| H8 | S546G, R547A, S550T | | Yes | 10^7^ | No | c87,BH97,I-41 | E2, E5 |
| **Engineered Potential N-linked glycosylation sites** | | | | | | | |
| H14 | 282 NDL -> NDS | | Yes | 10^7^ | No | BH15 | E1 |
| H23 | 282 NDL -> NDS, E398G | | Yes | 10^7^ | No | BH15 | E1 |
| H11 | 282 NDL -> NDS, 535 EHA ->NAT, | | Yes | 10^7^ | No | BH15,16DE6,c87, | E1, E2 |
|  | E398G, | |  |  |  | cl18,c8,I-41 |  |
| H16 | 403 KDN -> NDT | | Yes | 10^7^ | No | cl48,I-44 | E3 |
| H21 | 551 FYP -> NGT | | Yes | 10^7^ | No | c87,cl18,c8,I-41 | E2 |
| H20 | 590 SGG -> NGS | | Yes | 10^7^ | No | - | - |
| H22 | 590 SGG -> NGS, Y310T | | Yes | 10^7^ | No | I-29,BH141 | E4 |
| H27 | 590 SGG -> NGS, | | Yes |  | ND | ND | ND |
|  | 488 KEA->NAT,Y310T | | Yes |  |  |  |  |
| **Control** | | | | | | | |
| MVeGFP | | --- | Yes | 10^7^ | No | Neutralized by all mAb above |  |

* purchased human AB serum, sterile filtered, heat inactivated; ND, not done; TCID_50,_ 50% Tissue Culture Infectious Dose; R, complete or partial resistance

Table S1. MV-H# viruses with rationally designed mutations in potential immunodominant epitopes screened to identify monoclonal escape mutations
