# Supplementary material for: Epitope Dampening Monotypic Measles Virus Hemagglutinin Glycoprotein Results in Resistance to Cocktail of Monoclonal Antibodies
Source: PLoS One. 2013 Jan 3;8(1):e52306. doi: 10.1371/journal.pone.0052306 (PMC3536790; doi:10.1371/journal.pone.0052306)
Supplement: Table S2 — Mutations in MV-H# escape mutants delineate four different epitopes targeted by monoclonal antibodies. (DOCX) [file pone.0052306.s004.docx]

| **MV-H#** | **Rationally designed mutations** | **Escape mutations** | **mAb escape** | **Designated Epitope** |
| --- | --- | --- | --- | --- |
| MV-eGFP | none | - | - | - |
| MV-H14 | 282 NDL->NDS | 282 NDL->NDS | BH15 | E1 |
| MV-H11 | 282 NDL->NDS, 535 EHA->NAT, E398G | 282 NDL->NDS | BH15 | E1 |
|  |  | 535 EHA->NAT | 16DE6, I-41, c87 | E2 |
| MV-H5 | Q383N, A385G, K387R, G388A, | Not determined | I-44, cl48 | E3 |
|  | E395D, N396G, E398D |  |  |  |
| MV-H22 | Y310T, 590 SGG->NGS | Y310T | BH141, I-29 | E4 |
| --- | Previously published escape mutants | Y310D, L296I | BH38 | E4 |
|  |  |  |  |  |

Table S2. Mutations in MV-H# escape mutants delineate four different epitopes targeted by monoclonal antibodies.
