# Supplementary material for: Epitope Dampening Monotypic Measles Virus Hemagglutinin Glycoprotein Results in Resistance to Cocktail of Monoclonal Antibodies
Source: PLoS One. 2013 Jan 3;8(1):e52306. doi: 10.1371/journal.pone.0052306 (PMC3536790; doi:10.1371/journal.pone.0052306)
Supplement: Table S3 — Escape mutations against monoclonal antibodies that interfere with receptor binding are not confined to a single residue and can include a N-linked glycosylation site. (DOCX) [file pone.0052306.s005.docx]

| **mAb** | **Published escape mutations** | **Escape mutations in MV-δE4** | **Epitopes in MV-δE4** |
| --- | --- | --- | --- |
| I-29 | S313L or G314R | Y310C | E4 |
| I-41 | F552V | 535EHA->NAT | E2 |
| 16DE6 | G211S, G388D, S532F or R533G | 535EHA->NAT | E2 |
| I-44 | S189P, 187NCS->NCP | E395K | E3 |

Table S3. Escape mutations against monoclonal antibodies that interfere with receptor binding are not

limited to a single residue and can include a N-linked glycosylation site.
